# Electrical Spinal Stimulation, and Imagining of Lower Limb Movements to Modulate Brain-Spinal Connectomes That Control Locomotor-Like Behavior

**DOI:** 10.3389/fphys.2018.01196

**Published:** 2018-09-19

**Authors:** Yury Gerasimenko, Dimitry Sayenko, Parag Gad, Justin Kozesnik, Tatiana Moshonkina, Aleksandr Grishin, Aleksandr Pukhov, Sergey Moiseev, Ruslan Gorodnichev, Victor Selionov, Inessa Kozlovskaya, V. Reggie Edgerton

**Affiliations:** ^1^Pavlov Institute of Physiology, Russian Academy of Sciences, St. Petersburg, Russia; ^2^Department of Integrative Biology and Physiology, University of California, Los Angeles, Los Angeles, CA, United States; ^3^Institute of Fundamental Medicine and Biology, Kazan Federal University, Kazan, Russia; ^4^Department of Neurosurgery, Center for Neuroregeneration, Houston Methodist Research Institute, Houston, TX, United States; ^5^Velikie Luki State Academy of Physical Education and Sport, Velikiye Luki, Russia; ^6^Institute for Information Transmission Problems, Russian Academy of Science, Moscow, Russia; ^7^Russian Federation State Scientific Center, Institute for Bio-Medical Problems, Russian Academy of Sciences, Moscow, Russia; ^8^Institute Guttmann, Hospital de Neurorehabilitació, Institut Universitari adscrit a la Universitat Autònoma de Barcelona, Badalona, Spain; ^9^The Center for Neuroscience and Regenerative Medicine, University of Technology Sydney, Ultimo, NSW, Australia

**Keywords:** imaging, transcutaenous spinal cord stimulation, locomotor circuitry, brain-spinal connectome, TMS

## Abstract

Neuronal control of stepping movement in healthy human is based on integration between brain, spinal neuronal networks, and sensory signals. It is generally recognized that there are continuously occurring adjustments in the physiological states of supraspinal centers during all routines movements. For example, visual as well as all other sources of information regarding the subject's environment. These multimodal inputs to the brain normally play an important role in providing a feedforward source of control. We propose that the brain routinely uses these continuously updated assessments of the environment to provide additional feedforward messages to the spinal networks, which provides a synergistic feedforwardness for the brain and spinal cord. We tested this hypothesis in 8 non-injured individuals placed in gravity neutral position with the lower limbs extended beyond the edge of the table, but supported vertically, to facilitate rhythmic stepping. The experiment was performed while visualizing on the monitor a stick figure mimicking bilateral stepping or being motionless. Non-invasive electrical stimulation was used to neuromodulate a wide range of excitabilities of the lumbosacral spinal segments that would trigger rhythmic stepping movements. We observed that at the same intensity level of transcutaneous electrical spinal cord stimulation (tSCS), the presence or absence of visualizing a stepping-like movement of a stick figure immediately initiated or terminated the tSCS-induced rhythmic stepping motion, respectively. We also demonstrated that during both voluntary and imagined stepping, the motor potentials in leg muscles were facilitated when evoked cortically, using transcranial magnetic stimulation (TMS), and inhibited when evoked spinally, using tSCS. These data suggest that the ongoing assessment of the environment within the supraspinal centers that play a role in planning a movement can routinely modulate the physiological state of spinal networks that further facilitates a synergistic neuromodulation of the brain and spinal cord in preparing for movements.

## Introduction

One factor that has been shown to be important, particularly in movements such as locomotion, has been the degree of automaticity that is intrinsic to the neural networks that control stepping (Grillner, 2006; Rossignol and Frigon, 2011). Secondly, these networks have the ability to reorganize to rather dramatic functional levels when driven by activity-dependent mechanisms, which includes learning potential of spinal as well as supraspinal networks (Edgerton and Roy, 2009; Roy et al., 2012). Thus, mechanisms underlying the emergence of novel brain-spinal network connectomes to such activity-dependent transformations, have become an area of very high interest.

Functional magnetic resonance imaging has been proposed as a novel paradigm to study brain activation during walking (Jahn et al., 2004). Numerous studies have been performed focusing on the question of which areas of the brain are activated when imagining a given motor task compared to when actually performing the motor task. The general conclusion was that there were many areas the brain activated by both real and imagined movements (Deiber et al., 1998; Gerardin et al., 2000), but there, also, were some areas of activity that were unique to each condition (la Fougere et al., 2010). In this study and in other similar studies, there has been little effort to understand how the spinal circuitry may be neuromodulated when imagining-visualizing a motor task versus actually performing the motor task.

In the present study we examined the interactions of spinal and supraspinal networks in facilitating locomotor movements when the subject imagined rhythmic stepping movements in the presence of different intensities of stimulation or absence of transcutaneous electrical spinal stimulation. Also we characterized motor evoked responses in leg muscles induced by transcranial magnetic stimulation of motor cortex and/or by transcutaneous electrical spinal cord stimulation during actual stepping-like movements versus imagined movements. We hypothesize that the physiological state of the lumbosacral spinal networks that generate rhythmic stepping-like movements can be modulated by visual:imagining inputs projecting to selected combinations of spinal motor pools. These data suggest that the supraspinal and spinal networks synergistically function in a manner that facilitates the feedforwardness of complex movements such as locomotion and thus increases the likelihood of an intended movement to be performed successfully.

The central question in the present study is whether the impact of imagining-visualizing a motor task is mediated via spinal networks. This issue is becoming of increasing relevance given the recognition of the prominence of feedforward mechanisms among spinal networks when generating locomotor patterns (Gerasimenko et al., 2016). The design of the present experiments takes into account that in planning and execution of movements, in the end to execute well co-ordinated stepping all sources of input must be synergistic with the physiological state of the spinal networks that control the motor pools that will perform the movement.

## Materials and methods

Seventeen non-injured individuals participated in the study. The subject pool was of mixed gender (1 female, 7 males) and had a mean age of 32 ± 17 years (range 22–66). All subjects signed informed consent forms to participate in these experiments. The study was approved by the Human Subject Protection Committee at the Velikie Luki State Academy of Physical Education and Sport, Velikie Luki and conformed to the principles stated in the Declaration of Helsinki.

### Transcutaneous electrical spinal cord stimulation (tSCS)

A five-channel constant current stimulator (BioStim-5, Cosyma, Russia) was used (Grishin et al., 2017). Self-adhesive round electrodes (Lead-Look, Sandpoint, ID) with a diameter of 2.5 cm (cathodes) were placed on the skin between the spinous processes of the T11-T12 or L1-L2 vertebrae (hereafter referred to as T11 and L1) and two interconnected 5 × 9 cm self-adhesive electrodes (Axelgaard, ValuTrode Cloth) were placed over the iliac crests as anodes, as previously described elsewhere (Maertens de Noordhout et al., 1988; Gerasimenko et al., 2015). The stimulation waveform consisted of monophasic rectangular 1 ms pulses at a frequency of 30 Hz, each pulse filled with a carrier frequency of 5 kHz. The intensity of stimulation was adjusted sufficiently to generate involuntary rhythmic stepping-like movements without causing discomfort when delivered at T11 alone (range: 20–120 mA) during rest, that is when subjects were instructed to stay relaxed and not to resist or voluntary participate in the stimulation-induced motions. Stimulation at L1 was delivered as single 1 ms, monophasic, square-wave with frequency 0.3 Hz or manually to elicit spinally evoked motor potentials, hereafter referred to as sEMP, in leg muscles. Intensity of the stimulation ranged from 20 to 150 mA. Recruitment curves were constructed by plotting the magnitude of sEMP against increasing stimulation intensity.

### Transcranial magnetic stimulation (TMS)

TMS was delivered over the left primary motor cortex with single pulses using a Magstim Rapid^2^ stimulator (Magstim, Dyfed, UK). The double cone coil (110 mm of diameter) was placed parallel and approximately 1 cm lateral to the left from this intersection point (Knikou, 2014). At this coil position the intensity stimulation gradually increased to evoke motor evoked potentials, hereafter referred to as MEPs, in right tibialis anterior (TA) and medial gastrocnemius (MG) muscles. After eliciting and identification of these responses, the coil was fixed and maintained in that position during the experiment. To evaluate the recruitment curves of MEPs in leg muscles during TMS the intensity of stimulation gradually increased from 50 to 90% from maximal output. The cortically and spinally evoked motor potentials were recorded in the muscles of the right (upper) leg.

### Data recordings and analysis

Surface electromyogram (EMG) signals were recorded bilaterally using bipolar surface electrodes (MegaWin ME 6000 16-channel electromyography, Finland) placed bilaterally over the vastus lateralis (VL), medial hamstrings (HM), tibialis anterior (TA), and medial gastrocnemius (MG) muscles (Gerasimenko et al., 2010). EMG signals were differentially amplified (bandwidth of 10 Hz to 2 kHz) and digitized at a sampling rate of 2 kHz. The filtered EMG signals (bandpass filter with a low cutoff frequency of 30 Hz and high cutoff frequency of 200 Hz) were analyzed offline to eliminate the artifacts of stimulation. Angular movements of the right and left knee joints were recorded using goniometers attached laterally to the suspended legs.

**Figure 1:**
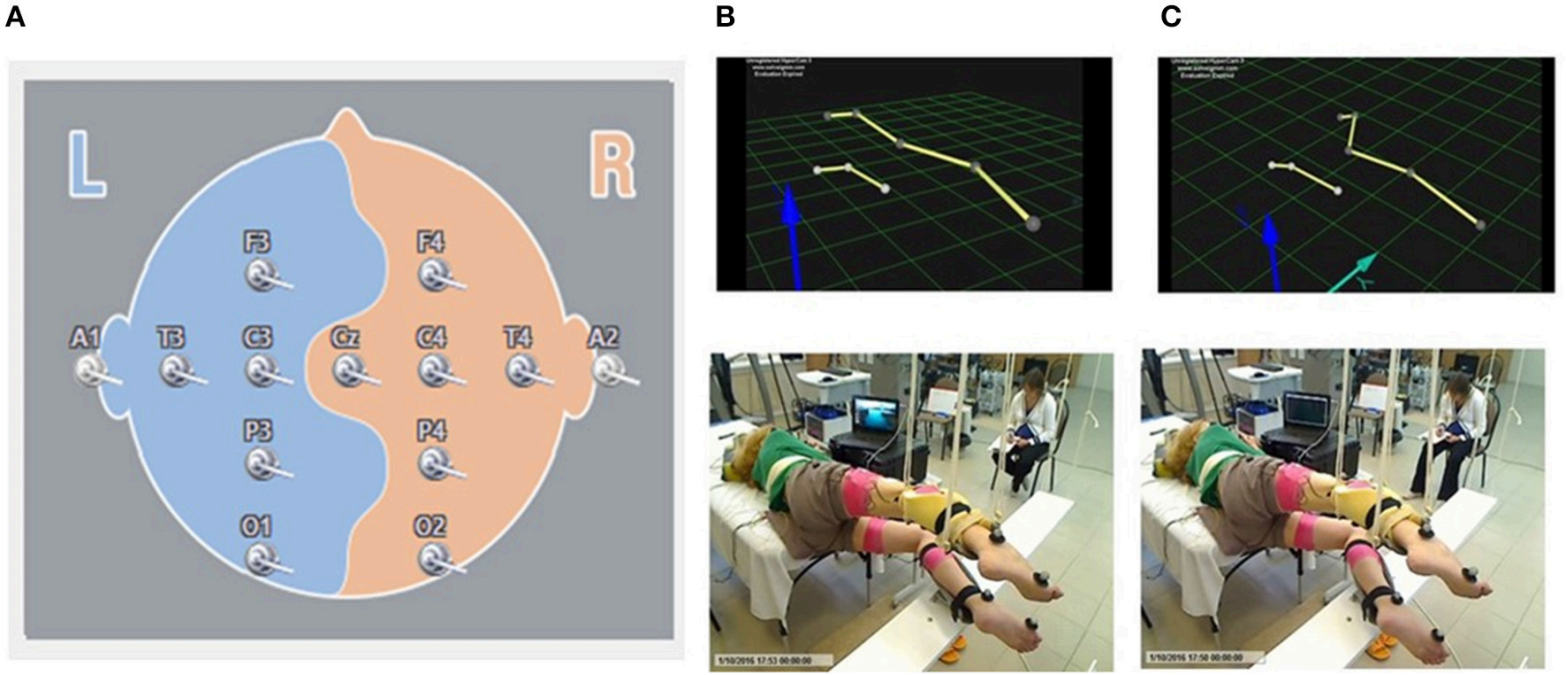
Localization of the surface EEG electrodes **(A)**. Position of the subject placed in gravity-neutral device. Visual imagery non-stepping (VI-NS) **(B)**. Visual imagery stepping (VIS) **(C)**.

EEG signal was recorded by surface electrodes, gathering data from 11 areas of the brain from the standard 10–20 scalp locations (Encephalan, Russia) (Figure 1A). Color topographic EEG maps of the brain for each of the frequency bands (Delta1 0.5–2 Hz, Delta2 2–4 Hz, Theta 4–8 Hz, Alpha 8–13 Hz, Beta 13–24 Hz, and Gamma 24–35 Hz) have been used. The spectral power of occipital, parietal, frontal and temporal areas has been calculated.

### Voluntary and imagined stepping

Initially the kinematic of movements using Qualisys motion capture system (Sweden) was recorded in one non-injured subject performing the voluntary stepping-like movements in gravity neutral device with comfortable stepping rhythm (about 0.6–0.7 Hz). Movements of the right (upper) and left (lower) legs were monitored using reflective markers placed at the lateral epicondyle of the humerus, greater trochanter, lateral epicondyle of the femur, lateral malleolus, and hallux. Then these movements were reconstructed in a stick figure. This stick figure was used for all subjects for visual imaginary stepping (VIS). The subjects were asked to imagine bilateral stepping while visually observing a stick figure presented on a video monitor which performed rhythmic stepping movements or was stationary (VI-NS) (Figure 1). The cortically evoked motor potentials (MEPs) and spinally evoked motor potentials (sEMP) in leg muscles were examined during voluntary stepping and during VIS when the subjects were placed in gravity-neutral device.

### Gravity-neutral device

Subjects were placed in gravity-neutral device when their legs were supported in a horizontal position as described previously (Gurfinkel et al., 1998; Selionov et al., 2009). Briefly, the subject was positioned on the left side with the right (upper) leg supported directly in the area of the shank and the left (lower) leg placed on a rotating brace attached to a horizontal board supported by vertical ropes secured to hooks in the ceiling.

### Experimental procedures

Several experimental conditions were alternated in a pseudo-random sequence for each individual and comprised: rest, voluntary stepping movements, and involuntary stepping movements induced by transcutaneous spinal cord stimulation (tSCS). Some combination of imagining: visual imagery stepping (VIS), no visual imagery stepping (NO-VIS, close monitor by paper) and visual imagery non-stepping VI-NS) were used. During movement induced by spinal stimulation, the subjects were instructed to relax and not to exert any effort to move or to resist the induced leg motions.

To determine the effect of visualizing and imagining generating stepping-like movements during spinal stimulation on rhythmic stepping movements we pre-selected a group of subjects. We initially tested 17 subjects using tSCS alone as well as VIS+tSCS on the ability to induce involuntary stepping movements. In our previous study we have shown that electromagnetic spinal cord stimulation or vibration of leg muscles were able to induce involuntary stepping movements ~10% of tested healthy subjects placed in gravity neutral position (Gerasimenko et al., 2010). Electrical transcutaneous spinal cord stimulation induced the involuntary stepping movements ~in 25–30% of tested healthy subjects (unpublished data). Present study inclusion-exclusion criteria for the subjects was their responsiveness to induce involuntary rhythmic stepping-like movements during tSCS or during VIS+tSCS. If this response was absent then this subject was excluded from the study. From the 17 subjects tested, eight subjects in which the spinal stimulation alone or in combination with imagining stepping initiated involuntary stepping movements were included for the study (Supplementary Video 1).

### Statistical analysis

Data are reported as means ± standard error of the mean (SEM). Overall significant differences among the variables studied during voluntary or passive stepping movements as well as during imagining stepping in the presence of transcutaneous spinal cord stimulation were determined using a one-way repeated-measures ANOVA with the level of statistical significance set at *P* < 0.05.

## Results

### Effects of motor imagining on locomotor behavior facilitated by spinal stimulation

**Figure 2:**
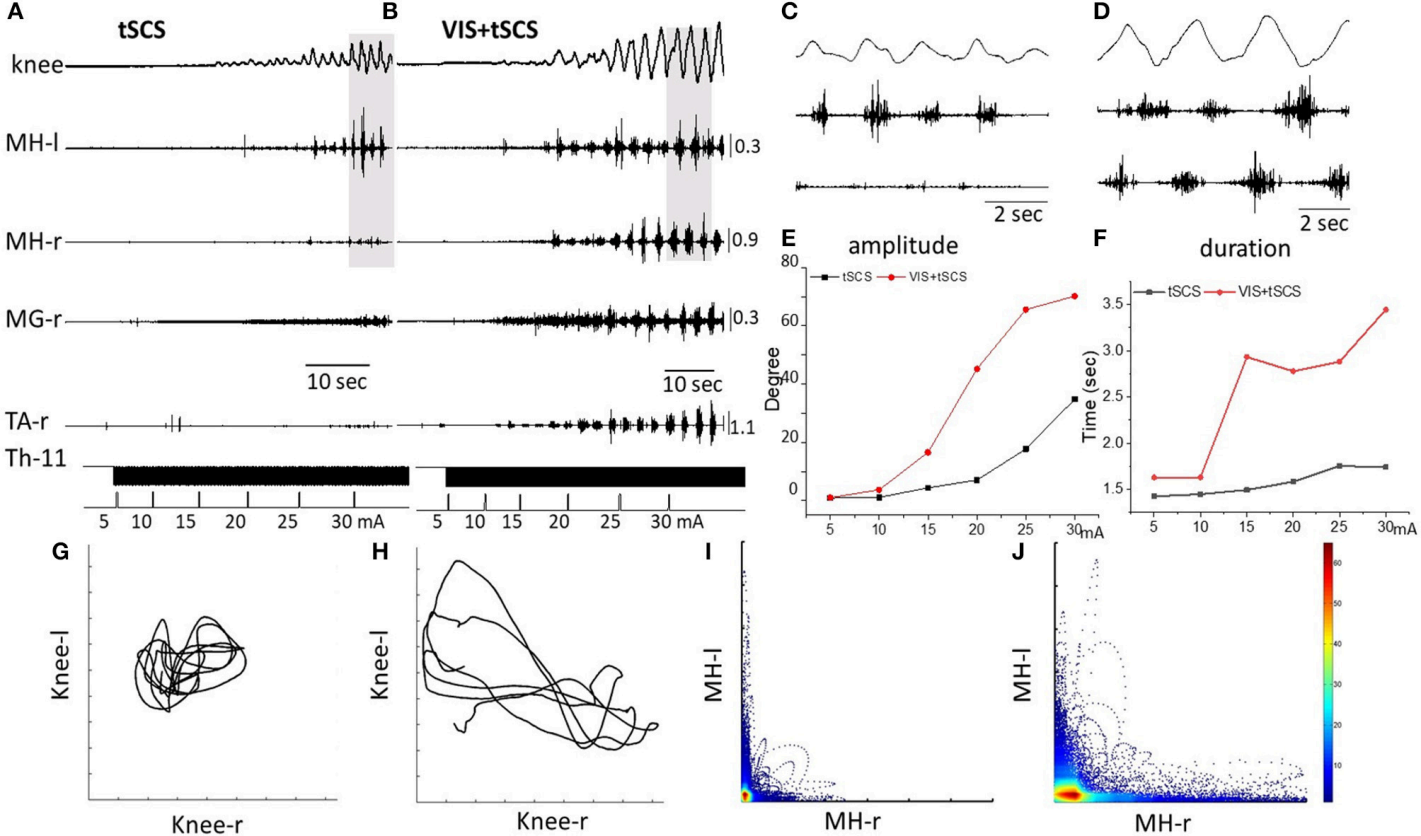
Angular excursions of the right knee joint and EMG activity in the medial hamstring (MH) in right and left legs, right medial gastrocnemius (RMG), and right tibialis anterior (RTA) during gradual (by 5 mA) increasing intensity of spinal stimulation alone **(A)** and in the presence of visual imagery of stepping (VIS) **(B)** in Subject D.G. Knee excursion and EMG bursts marked by a gray background **(A,B)** are displayed with an extended time scale in **(C,D)**, correspondingly. Plots of amplitude displacements **(E)** and cycle period **(F)** of knee joint during tSCS alone and VIS+tSCS. Kinematics coordination based on knee (left) and knee (right) movements during tSCS **(G)** and VIS+tSCS **(H)**. Pattern of reciprocity for EMG activity of the HM (left) and HM (right) during tSCS **(I)** and VIS+tSCS **(J)**. EMG calibration: mV.

At the first stage of the study the threshold of spinal stimulation to induce involuntary stepping movements in subjects placed in gravity-neutral device has been determined. Gradually increasing of intensity of tSCS applied at T11-T12 vertebrae (30 Hz) elicited stepping in five of seven subjects tested. The threshold for inducing the stepping was subject-dependent and ranged from 20 to 60 mA. The rhythmic movement patterns induced by tSCS were reflected by the EMG bursting activity in the right and left thigh muscles and in the kinematics of the right knee (Figure 2A).

When the subject was imagining performing the task as shown by the stick figure and visualized by the subject involuntary stepping movements were facilitated during tSCS in all subjects (Supplementary Video 1). Spinal stimulation in the presence of VIS initiated stepping movements with greater excursions and EMG burst amplitudes than were with stimulation at T11 alone (Figure 2B). Bursting EMG patterns were observed not only in proximal but also in distal leg muscles, compared to proximal muscles only during tSCS alone (Figure 2B). The amplitude of knee joint excursion (C) and cycle duration (D) were greater during VIS+tSCS than with tSCS alone (Figure 2). For every 5 mA increase in stimulation intensity from 5 to 30 mA the amplitude in knee excursion increased (Figure 2E). In the VIS+tSCS the amplitude in knee excursion was greater than with tSCS alone at each intensity stimulation on 114, 338, 378, 638, 370, 213%, correspondingly.

Generally, the combined VIS+tSCS resulted in larger flexion-extension movements and a more rapid increase in the amplitude of knee excursions. The cycle period also increased depending on intensity of tSCS. During VIS+tSCS we observed interlimb coordination. There was a clear pattern of reciprocity in the modulation of EMG amplitudes in the left and right HM (Figure 2J) and left vs. knee angular displacements (Figure 2H).

**Figure 3:**
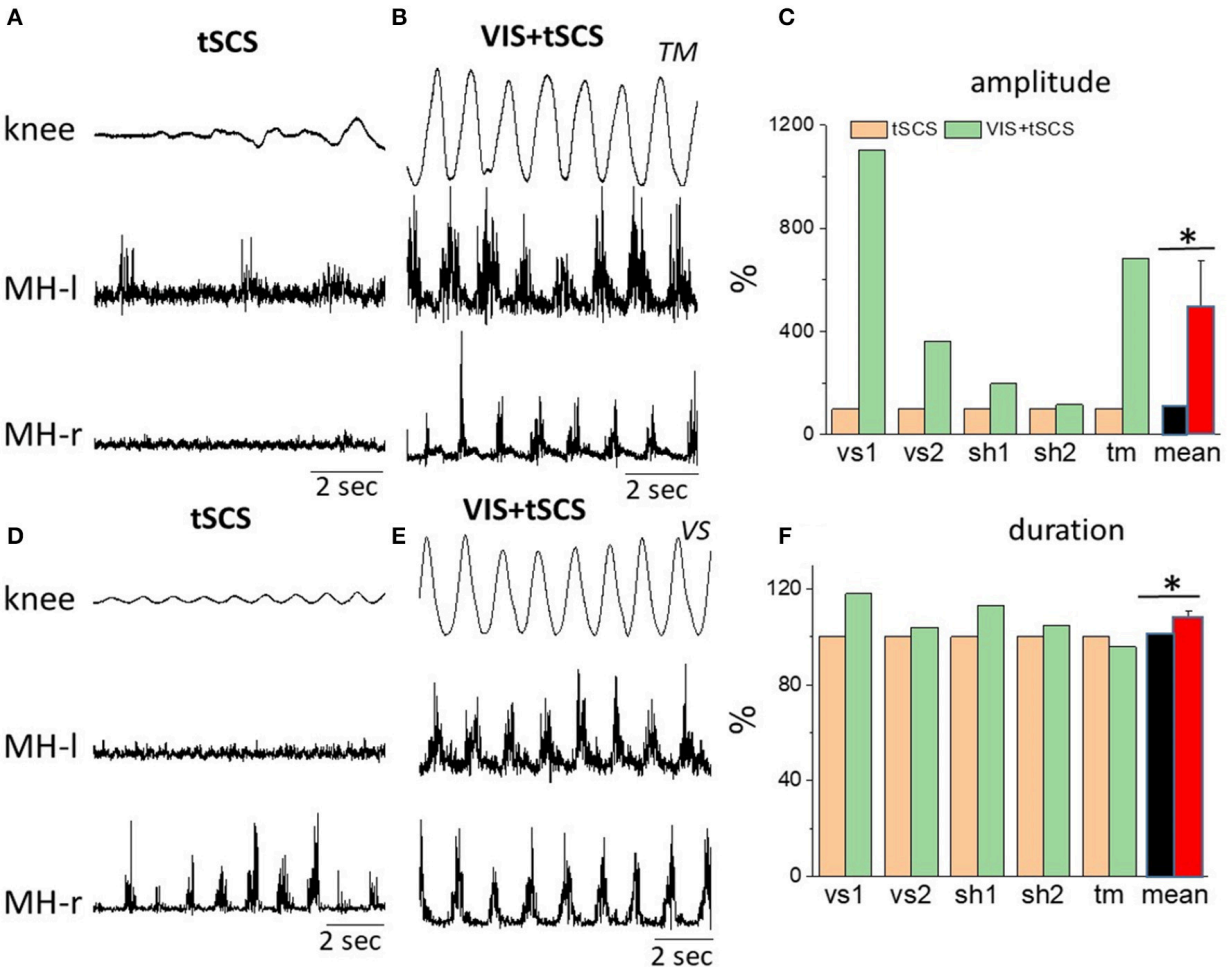
Angular excursions of the right knee joint and corresponding rectified EMG activity in the left and in the right hamstrings when stimulating at T11,30 Hz **(A,C)** and when combining with imagining stepping **(B,D)** in subjects *TM* **(A,B)** and *VS* **(D,E)**. **(C)** Mean peak-to-peak excursion amplitudes (% of tSCS alone) at the knee joint and the corresponding cycle period durations **(F)** are shown for the 8–10 step-like movements in a gravity-neutral apparatus in response to stimulation at T11 only and at VIS+tSCS for three subjects: vs, sh, and tm (subject vs and sh were tested twice). *Significantly different at *p* < 0.05.

Figure 3 shows the kinematic of knee joint movements and EMG activity of left and right hamstrings during 8–10 step-like movements induced by tSCS (30 Hz) at optimal intensity applied at T11 alone vs. tSCS (with the same parameters) in combination with VIS. The raw EMG and goniometry data demonstrate the complementary effects of spinal stimulation and VIS. In some subjects the amplitude of angular knee excursions during VIS+tSCS increased (6–10 fold) as compared to tSCS alone, when the knee angle changed insignificantly (Figure 3).

**Figure 4:**
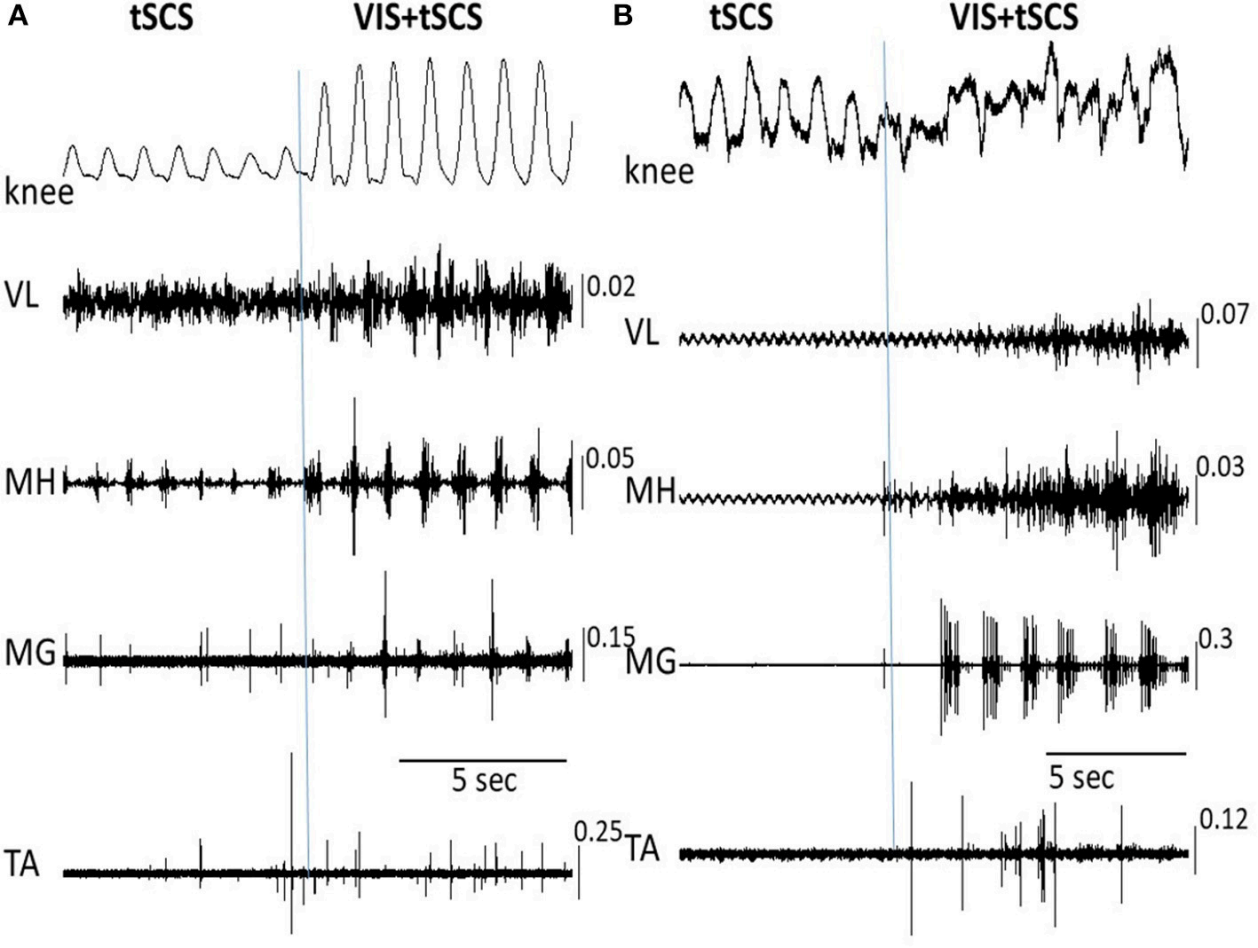
Angular excursions of the knee joint and corresponding EMG activity in the vastus lateralis (VL), medial hamstring (MH), medial gastrocnemius (MG), and tibialis anterior (TA) muscles during tSCS alone at T11 and VIS+tSCS in two subjects **(A,B)** are shown. tSCS was initiated and VIS was subsequently added (indicated by vertical line). In subject **(B)** the VIS+tSCS generated a robust, but highly erratic motor output, with each motor pool generating qualitatively different EMG patterns. EMG calibration: mV.

We next tested the effects of sequentially adding VIS with stepping performance that had already been initiated by spinal stimulation. Delivering VIS in the presence of spinal stimulation produced more robust stepping movements and higher amplitude bursting EMG patterns, both in proximal and in distal leg muscles (Figures 4A,B). Adding VIS to T11 (30 Hz) stimulation immediately resulted in enhancing the kinematics as well as the EMG patterns and involved in the movement of the ankle joint as reflected by activation of the medial gastrocnemius and tibialis anterior (Figures 4A,B). The addition of VIS to spinal stimulation facilitated the stepping performance in all subjects, while in some subjects the integration of supraspinal and spinal sources of neural influence on stepping was more complex resulting in generation of an irregular stepping pattern (Figure 4B). This example, in Figure 4B, the EMG pattern of bursting of the MG motor pool clearly remained rhythmic, but the rhythmicity of the VL, MH, and TA motor pools was much less.

**Figure 5:**
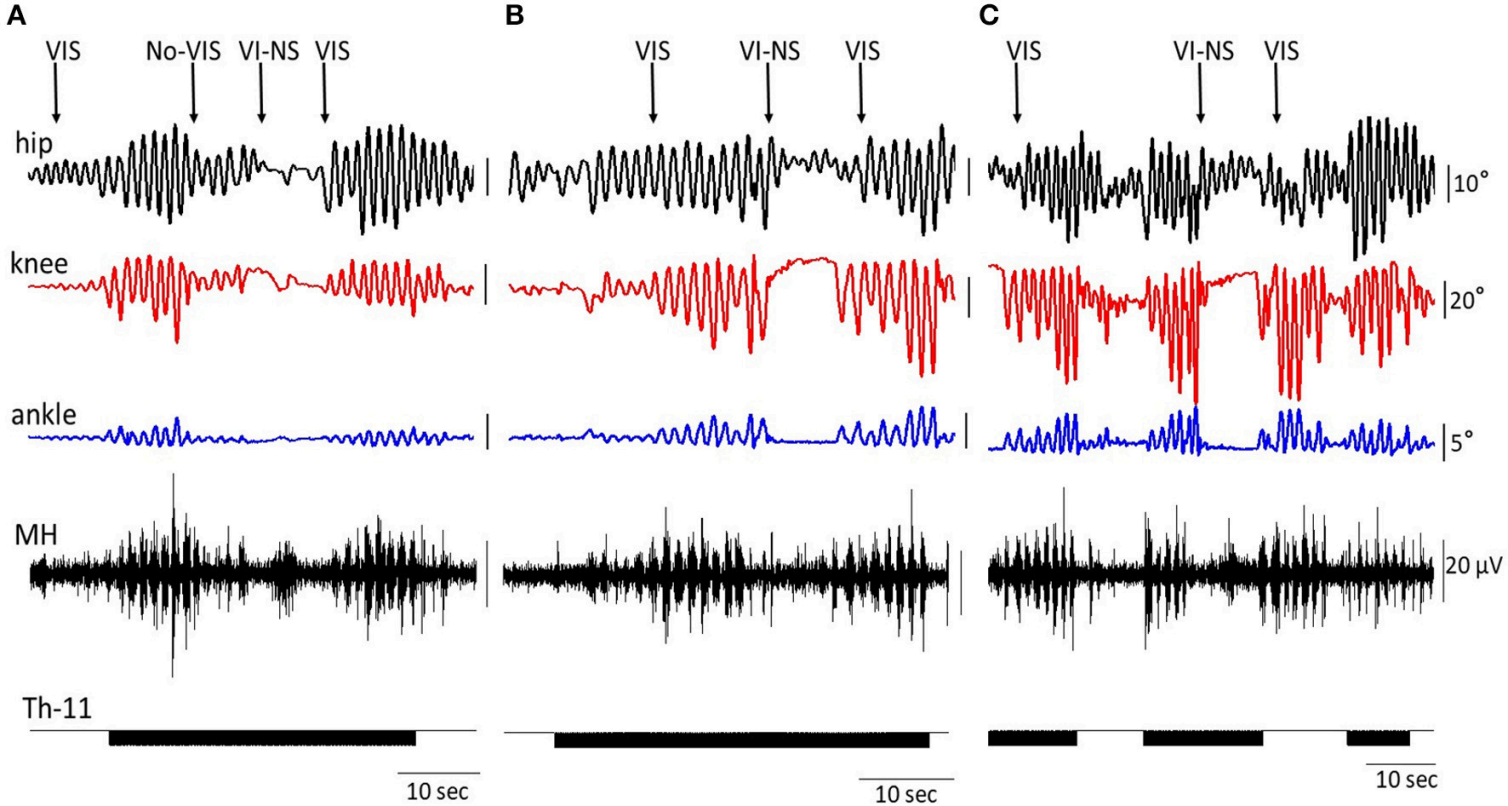
An example of the on-off effects of visual imagery of stepping (VIS) and visual imagery of non-stepping (VI-NS) and/or spinal stimulation on the excursion at the right knee joint and the EMG activity in the hamstring. Effects of VIS, VI-NS, and stop imagining stepping (No-VIS), i.e., not looking at any image are shown. Data from same subject, same session **(A–C)**.

Figure 5 shows the on-off effects of when the subject is viewing four different visual fields to imagine performing bilateral stepping when it is being induced by tSCS at T11 and also when the oscillation was initiated with VIS. During VIS, tSCS applied to T11 effectively initiated stepping movements (Figure 5A). Replacement of VIS immediately with no image (No-VIS) immediately reduced the movement by subsequently reinserting the VI-NS condition the oscillations were terminated until the VIS condition was presented. The stepping performance consistently reflected the excursions of the knee joint and bursting EMG amplitude (Figure 5A). Replacement of VIS on non-stepping visual imagery (VI-NS) practically stopped stepping, and subsequent replacement of VI-NS on VIS recovered stepping performance (Figures 5A,B). When tSCS was stopped the stepping movements mediated by VIS+tSCS was terminated as well, but tSCS immediately re-activated stepping movements (Figure 5C). Interestingly, the termination of tSCS during replacement of VI-NS with VIS resulted in initiation of stepping movements (Figure 5C). It is noteworthy that replacement of a VI-NS with VIS in the presence of tSCS, immediately recovered coordinated stepping movements in hip, knee and ankle joints (Figures 5A,B).

**Figure 6:**
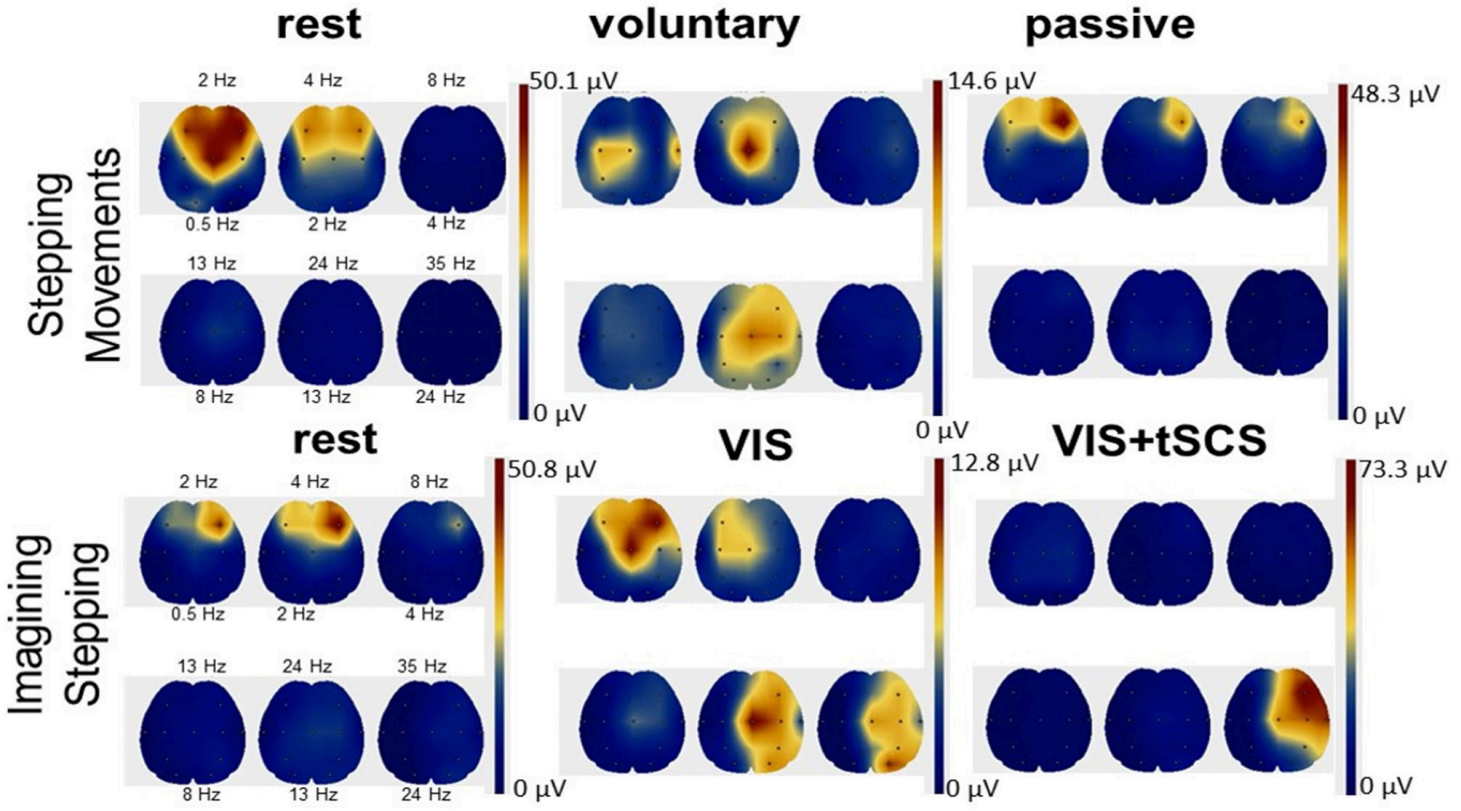
Scalp topographic mapping of the frequency components of EEG activity during the rest, voluntary, and passive stepping performance, as well as during visual imagery stepping (VIS) in the presence and the absence of tSCS.

EEG rhythms during voluntary and passive stepping-like movements in the subjects placed in gravity neutral device, as well as during VIS or VI-NS conditions were recorded. One of the main points of these recordings was the verification that the subjects generated a relative similar unique EEG pattern for the different experimental conditions, for example, when resting, imagining, and visualization of rhythmic stepping-like patterns the frequency components of EEG during voluntary stepping were in the range: 0.5–4 Hz (δ) and 13–24 Hz (β), whereas during imagination of the stepping together with these components an additional component 24–35 Hz (γ) appeared (Figure 6). During tSCS the component 24–35 Hz (γ) was dominating (Figure 6).

### Cortically and spinally evoked motor potentials

**Figure 7:**
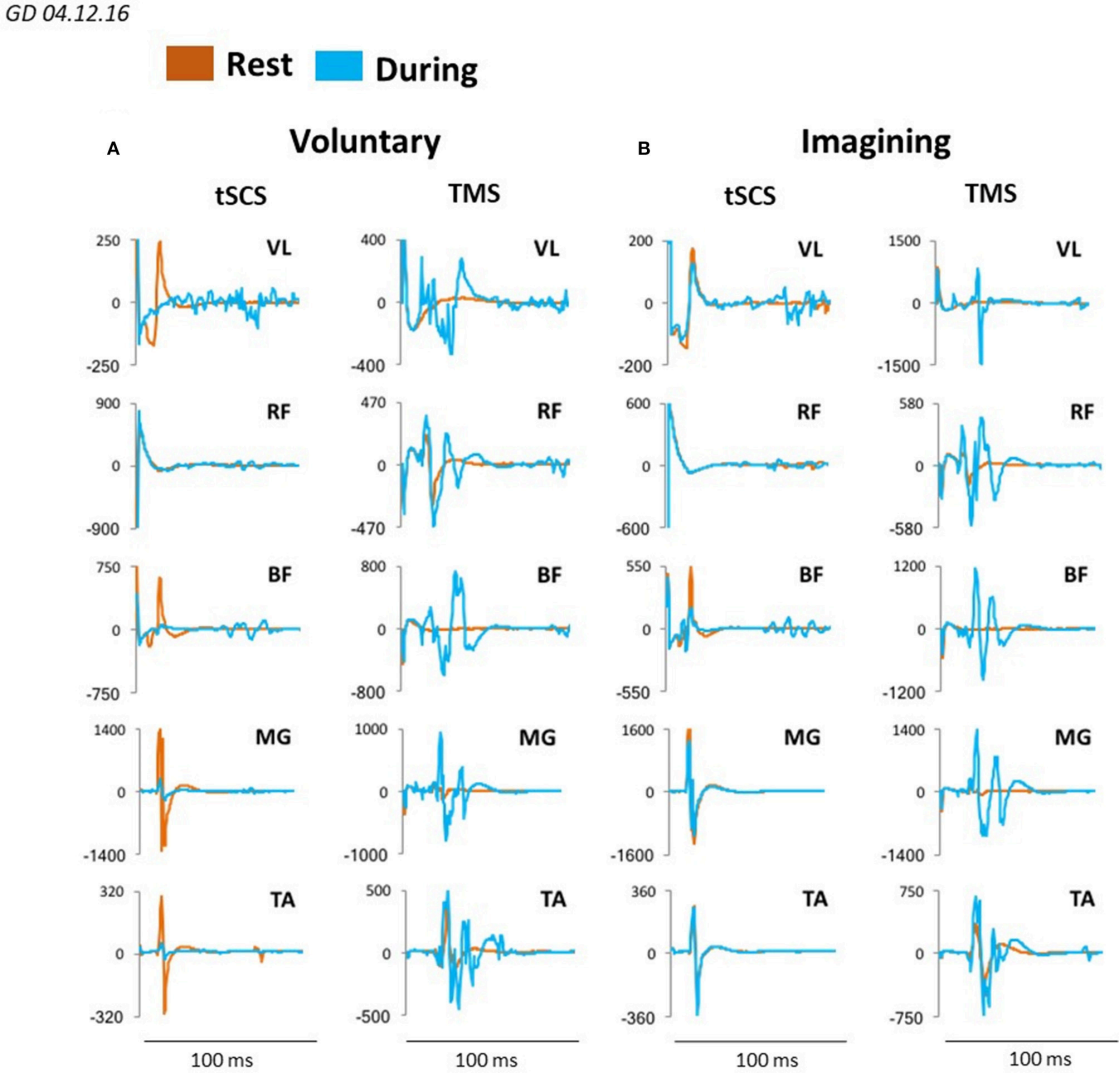
Averaged sEMP during tSCS delivered at L1 and MEPs during TMS, recorded in mm. vastus lateralis (VL), rectus femoris (RF), medial hamsting (MH), medial gastrocnemius (MG) and tibialis anterior (TA) in one participant at rest (orange) and during voluntary stepping (blue) **(A)** as well as during visual imagery stepping (blue) **(B)**.

We evaluated the motor responses in leg muscles induced by tSCS applied to L1 and by TMS in subjects placed in gravity neutral device during voluntary stepping, as well as during VIS. During VIS, as well as during real stepping, TMS or tSCS were delivered randomly within the stepping cycle. sEMP in leg muscles to L1 stimulation (0.3 Hz) as well as MEPs to TMS (0.1 Hz) were examined. The stimulation intensities were adjusted to produce half-maximal motor potentials in distal (TA and MG) leg muscles to TMS and/or tSCS. These motor potentials obtained at rest (control) were compared with analogous motor potentials recorded during voluntarily generated stepping performance or during VIS. We have found that during voluntary movements as well as during VIS the sEMP in the leg muscles were inhibited, whereas MEPs were facilitated. Compared to the resting state MEPs were facilitated during both voluntary stepping in 0G conditions and during VIS while, relative to the rest, sEMP were inhibited during stepping for each of these conditions (Figure 7).

**Figure 8:**
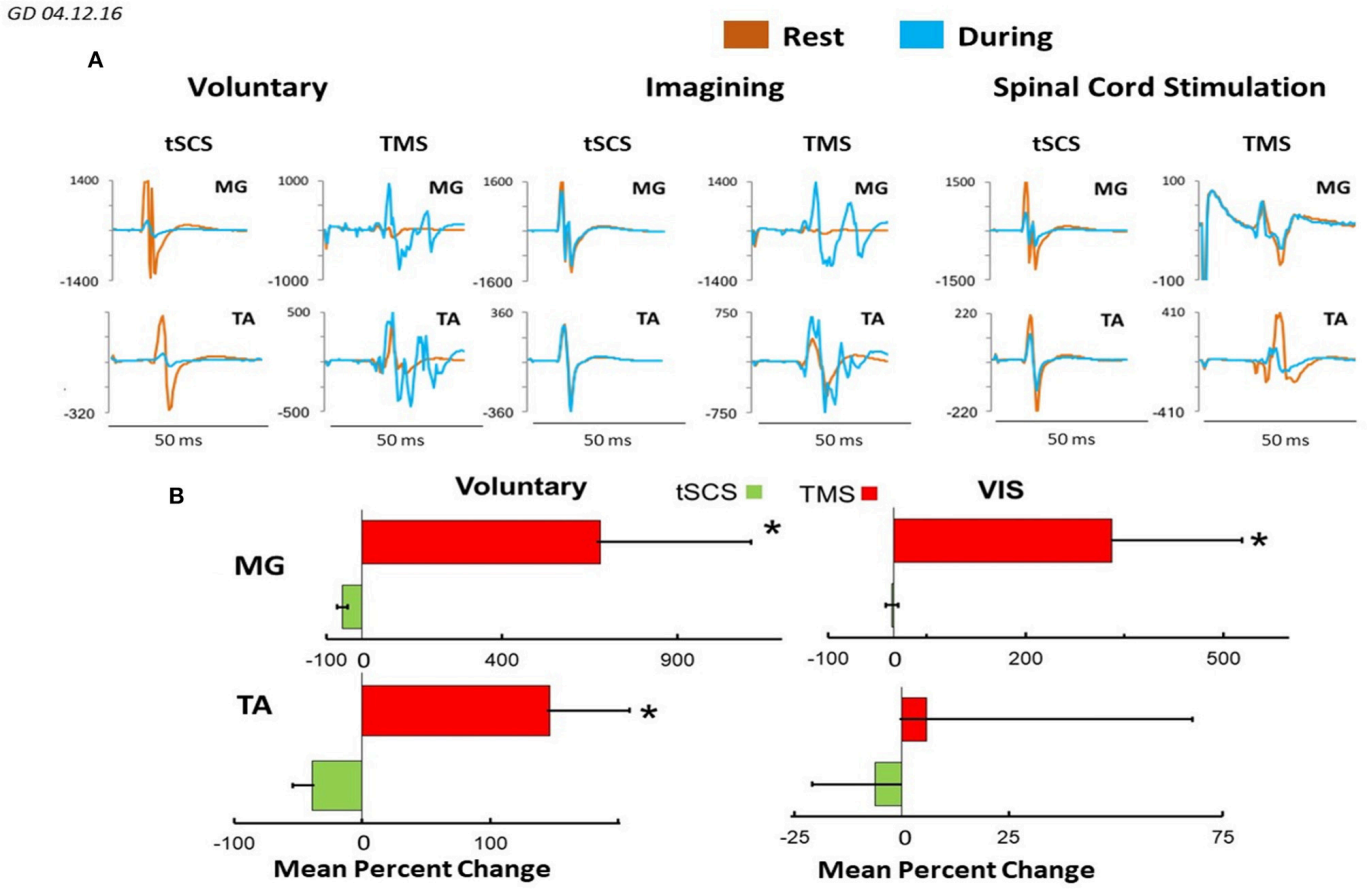
**(A)** Mean ± SEM (*N* = 7 for SCS, and 5 for TMS), **(B)** mean 4–6 evoked responses per subject) percent changes of peak-to peak amplitude relative to the rested state, of the motor evoked responses in MG and TA muscles in response to TMS and tSCS at a stimulation (L1) during: voluntary stepping movements, and visual imagery stepping (VIS). *Significantly different at *p* < 0.05.

Figure 8 demonstrates plots mean (*N* = 7 subjects) percentage change of peak-to peak amplitude of motor responses in MG and TA induced by TMS or by tSCS (L1) during different motor tasks. In MG mean percent (relative to the rest) of the facilitation of TMS induced MEPs during voluntary stepping was 680% while during imagining was 331%. The inhibition of sEMP in this muscle to L1 was correspondingly stronger during real stepping than during imagining stepping (−54 and −2%, correspondingly). In TA, the facilitation of MEPs was also observed during real stepping (146%) and during imagining (5%) but the degree of the facilitation was significantly lesser than in MG. At the same time the inhibition of sEMP during execution of these motor tasks (−39% and −6%) in TA was stronger than in MG. Continuous tSCS delivered at T11 (30 Hz) facilitated TMS induced MEPs in MG (261%) and TA (20%) and inhibition of L1 induced sEMP in these muscles (5 and −16%), correspondingly (Figure 8).

In the next series of the experiments on the same subjects but 3 weeks later we evaluated cycle-dependent neuromodulation of TMS and tSCS evoked responses in leg muscles during execution of the same motor tasks. For this aim the TMS and/or tSCS stimulation was applied during knee flexion or knee extension in the same points of knee excursions. During VIS, the single tSCS at L1 was triggered manually in according to the timing of the stick figure stepping during knee flexion or knee extension visually observed on a video monitor.

**Figure 9:**
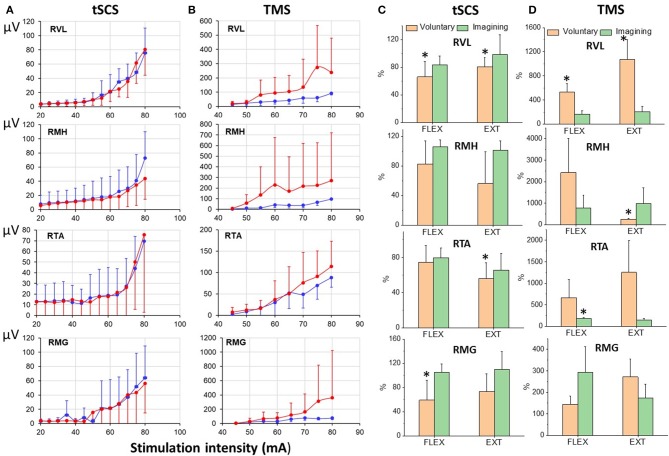
**(A)** Recruitmentcurves of sEMP to L1 stimulation and **(B)** recruitment curves of MEPs to TMS (mean ± SEM) in right leg muscles at rest (blue) and during VIS (red) (*N* = 6 subjects). Percentage of changes of peak-to peak amplitude of the motor evoked responses (relative to rest) induced during knee flexion or knee extension during voluntary stepping and during VIS. RVL, right vastus lateralis; RMH, right medial hamstrings; RTA, right tibialis anterior; RMG, right medial gastrocnemius muscles. *Significantly different at *p* < 0.05.

Figure 9 illustrates the difference in recruitment curves for proximal (RVL and RMH) and distal (RTA and RMG) leg muscles in response to tSCS at L1, or to TMS delivered over the left motor cortex for participant at rest and during imagining of stepping. The intensities of the stimulations were increased gradually to reach maximal magnitude of the responses. Recruitment curves (mean = 6 subjects) for sEMP during rest and during VIS differed insignificantly showing the tendency for inhibition of the responses during imagining (Figure 9A), whereas recruitment curve for MEPs the facilitation of the motor responses during imagining (Figure 9B).

The modulation of tSCS- and TMS-evoked motor responses during voluntary stepping in subjects placed in gravity-neutral device was cycle-dependent (Figure 9C). As in our prior studies the sEMP during voluntary stepping, as well as during imagining of stepping, were inhibited relative to the rest but nevertheless they were cycle-dependent modulated during voluntary stepping (Figure 9C). TMS induced MEPs, like in studies described above, were significantly facilitated during voluntary stepping and during imagining of stepping and also were cycle-dependent during voluntary stepping (Figure 9D). During VIS we did not observe clear cycle-dependent modulation of neither sEMP nor MEPs in the leg muscles.

## Discussion

Based on the present data derived from uninjured subjects it seems rather clear that imagining-visualizing a given motor task modulates the physiological state of spinal networks, which in effect prepares in a feedforward manner the spinal networks that will generate a motor task. This finding in the present study is that a very complex and dynamic neural network can be transformed in and out and to different levels of locomotor states by imagining-visualizing a stick figure in a non-stepping or a stepping state.

### Are synergistic interactions of supraspinal and spinal networks controlling agonist-antagonist motor pools critical?

In the present study we observed different degrees of synergism among the motor pools that drive rhythmic stepping-like movements. The level of electrical current necessary to initiate rhythmic stepping is significantly lower when the subject is imagining stepping while visualizing a rhythmic stepping figure (Figure 2). The decrease of motor threshold of TMS-induced responses during motor imagery, is consistent with observation of Li et al. (2004). This facilitating effect of visualizing and imagining a stepping figure was also revealed when comparing the amplitude of stepping initiated by tSCS alone compared with that initiated by tSCS at the same current level plus visual imagery of stepping (Figures 3, 4A,D). In one subject, however, on multiple occasions, when the VIS was added after a robust rhythmic pattern had already been initiated with tSCS, the movement of the legs became erratic, but highly active, with a clear rhythmic pattern returning when VIS was terminated. But even in this case there was a selective effect with some motor pools showing that erratic EMG pattern while other motor pools were more rhythmic in spite of the erratic motor pattern (Figure 4C). This special case perhaps, demonstrates a particular principle of controlling stepping that reflects the continuous and extensive interactions from which decisions must be made that weigh in real time the relative impact of different sources of inputs that drive the relative level of activation of neuronal networks that drive each motor pool. It can be suggesting that imagining not only increased the excitability of neuronal locomotor-related network but increased sensitivity for peripheral feedback input as well. This dynamic process of sensory-motor interaction and integration of multiple inputs must drive the combination of motor pools that will define the movement. Similar effects of sensory-motor interaction were observed during vibration-induced stepping. Sometimes during vibration the limbs transition spontaneously from forward to backward and again to forward stepping movements can occur (Selionov et al., 2009).

The dynamic interaction of supraspinal and spinal networks is present even more clearly in Figure 5C. Over the course of different combinations of effects of tSCS, as well as stepping in the presence of VIS or VI-NS, demonstrates that the timing and/or order of presence and even the origins of these inputs play an important role in the magnitude of the rhythmic movements. In the translation from a non-stepping state (VI-NS) to a stepping state (VIS) in the presence of tSCS, an immediate simultaneous generation of highly coordinated rhythmic stepping in the hip, knee and ankle occurs. In virtually every combination of variables studied VIS facilitated coordinated stepping patterns of locomotor networks.

### Electrophysiological evidence of imagining and visualizing stick figure stepping

To determine the effect of different sources of input to the “final” common network of a motor pool, we compared the amplitudes of evoked responses in response to TMS and to single pulse stimulation at L1 when the subject was resting, receiving a 30 Hz tSCS to induce lower limb rhythmic movements, and during voluntary rhythmic stepping movement. The general conclusion from these experiments is that the greatest facilitating effect on TMS evoked responses occurred during voluntarily driven rhythmic movements, with the magnitude of the VIS effect being less than the voluntary effect in both the MG and TA (Figures 7, 8). This suggests that imagining stepping had a facilitatory effect independent of the excitatory input derived from cutaneous and proprioceptive input associated with the voluntary and tSCS-induced limb movement. The VIS effect on the TMS induced MEPs compared to rest was evident over a range of TMS intensities for every motor pool studied but not with tSCS single pulse stimulation currents (Figures 9A,B). Another detail of the modulatory properties of interest is that the amplitude of the TMS evoked responses was step-phase dependent during voluntary stepping. As might be expected, there was no indication of phase-dependent effects of VIS on TMS induced responses given that the legs were not moving in this case (Figure 9D). Combined, these observations seem to suggest that the facilitating effect of imagining can be rhythmic if the visual input is rhythmic but if static, it may facilitate a non-rhythmic, tonic effect. The voluntary rhythmic movement could be derived from a consciously perceived motor drive and further assisted by proprioceptive and cutaneous drives.

### Implications of observations to the control of movement

We have found that cortically evoked MEPs in leg muscles were facilitated during execution of different motor tasks: voluntary movements, VIS, in the presence of tSCS at T11 (30 Hz), whereas L1 induced sEMP during these motor performances were inhibited (Figure 8). The present results demonstrate that the amplitude of MEPs recorded from proximal and distal muscles and flexor and extensor muscles when stimulating the motor cortex with the same fixed TMS parameters varied widely across experimental conditions. These experimental conditions were designed to modulate the prominence of consciously controlled input, visually driven input and proprioceptive input on spinal networks that drive rhythmic stepping-like movements. Under these conditions the efficacy of a given stimulus initiated at the motor cortex can vary as much as sevenfold, depending on the level and sources of other inputs projecting to these spinal locomotor networks. The amplitude of the spinally tSCS and cortically TMS-induced responses can be viewed essentially as a quantitative estimate of the magnitude of the net modulatory drive on the population of synapses of the spinal network projecting to each muscle when there is and when there is no rhythmic movement. This interpretation is based on the detailed analysis by Bodine et al. (1987) that the relationship of the forces generated by a motor unit is determined largely by the number of muscle fibers (thus proportional to action potential amplitude) per motor unit and the consistency of the order of recruitment within a motor pool. The highly nonlinear relationship between the stimulation intensity and the amplitude of the SEMPs shown in Figure 9 in response to tSCS matches well with that which would be predicted based on the motor units that are activated at the lower stimulation strengths are considerably smaller, i.e., innervate fewer muscle fibers, with there being larger motor units being activated at the higher strengths of stimulation. The impressive result is that VIS seems to have the capacity to modulate the net excitability level of motor pools to about 40% of that observed with voluntary stepping (see Figures 8B, 9B). This compares to about 10% when the limbs are induced to generate a stepping-like motion with tSCS, which probably reflects the level of excitability derived from proprioception. We suggest that this phenomenon reflects a strategy for motor control that may be far more important than generally recognized. More specifically, this effect can be viewed as one which prepares the spinal networks projecting to a given combination of motor pools and thus the muscles necessary for the movement intended, therefore serving as a spinal feed-forward mechanism as discussed previously (Gerasimenko et al., 2016).

### How does the spinal excitability change during imagining stepping?

It is well known that the same brain structures have been activated both during actual movements and during imagining movements (Guillot et al., 2012; Hétu et al., 2013; Kraeutner et al., 2014; Xu et al., 2014). However, there are some brain structures which are active during actual movements but not active during imagining movements. It seems that during actual movements some brain structures may be activated via proprioceptive and cutaneous afferents projecting to brain networks, whereas during imagining movements the peripheral feedback is absent (Di Rienzo et al., 2014). Another explanation is that during imagining the inhibitory effects associated with motor commands may prevent the actual generation of movement (Guillot et al., 2012). It has been suggested that during imagining the inhibition of motor commands can have different origins, including spinal cord (Rieger et al., 2017). Some authors suggest that imagining movements are accompanied by generations of motor commands activating the spinal interneurons but without activation of alpha-motoneurons (Grosprêtre et al., 2016). There are contradictory data regarding to the modulation of spinal excitability during imagining. Facilitation of the monosynaptic reflex (H-reflex) during motor imagery (Cowley et al., 2008) as well as the inhibition of the H-reflex has been reported (Oishi et al., 1994). Recently it was shown that the facilitatory effect of motor imagery on spinal reflex excitability is bidirectoral: from upper limb to the lower limb and vice versa. It means that spinal facilitation does not correspond to the imaginary involved muscles (Nakagawa et al., 2018).

The general conclusion from our electrophysiological evoked potential data is the following. sEMP in proximal and distal leg muscles generated by stimulation at L1 in subjects placed in gravity neutral device were inhibited (relative to resting- not stepping) both during voluntary and/or tSCS induced stepping movements as well as during imagining movements (Figures 7–9). The mean (*N* = 7) percent of inhibition (relative to the rest) of sEMP in MG and TA was −54.6 ± 29.9 and −39.1 ± 38.9, correspondingly during voluntary stepping movements and −2.86 ± 19.3 and −6.31 ± 39.5 during imagining stepping (Figure 8). The modulation of these inhibited potentials were step phase dependent both in proximal and distal leg muscles during voluntary stepping movements, but not during imagining stepping.

## Conclusion

The primary conclusion from the present experiments is that the supraspinal and spinal networks that drive rhythmic stepping in *in vivo* are constantly changing their physiological state to accommodate the programs planned by the nervous system. Supporting this conclusion is the observation that when imagining performing a stepping motor task while visualizing a rhythmically stepping stick figure significantly increases the amplitude and power of the locomotor network even with no voluntary effort to step. This means that the spinal interneuronal projections to multiple motor pools can be facilitated and co-ordinate by imagining stepping as seen visually from a lower limb stick figure. The magnitude of this visual-imagining effect is a function of the tonic sub-motor threshold at any given instant. Thus, we hypothesize that spinal interneurons linked to locomotion play a major role in interpretation and final decision-making as to how to react to any given ensemble of inputs that are continuously originating from multiple sensory systems. One final implication of the present study is that the supraspinal and spinal interneurons seem to form functionally seamless networks that are continuously preparing for subsequent events. That is, the highly detailed feed-forwardness of the spinal neurons is well prepared for the “planned” action to be performed.

## Author contributions

YG performed experiments, conceptualized the study, analyzed the data, and wrote the initial manuscript. DS and PG performed experiments, analyzed the data, wrote, and edited the manuscript. JK analyzed the data. TM, AG, AP, SM, RG, VS, and IK performed experiments. VE performed experiments, conceptualized the study, wrote, and edited the manuscript.

### Conflict of interest statement

VE, YG, and PG researchers on the study team hold shareholder interest in NeuroRecovery Technologies and hold certain inventorship rights on intellectual property licensed by the Regents of the University of California to NeuroRecovery Technologies and its subsidiaries. The remaining authors declare that the research was conducted in the absence of any commercial or financial relationships that could be construed as a potential conflict of interest.

## Supplementary material

Supplementary Video 1 Video demonstrating effects of TSCS only and VIS+TSCS in 6 subjects tested in the gravity neutral device.
